# Intrinsic plasmids influence MicF-mediated translational repression of *ompF* in *Yersinia pestis*

**DOI:** 10.3389/fmicb.2015.00862

**Published:** 2015-08-21

**Authors:** Zizhong Liu, Haili Wang, Hongduo Wang, Jing Wang, Yujing Bi, Xiaoyi Wang, Ruifu Yang, Yanping Han

**Affiliations:** ^1^State Key Laboratory of Pathogen and Biosecurity, Beijing Institute of Microbiology and EpidemiologyBeijing, China; ^2^Animal Husbandry Base Teaching and Research Section, College of Animal Science and Technology, Hebei North UniversityZhangjiakou, China

**Keywords:** *Yersinia pestis*, sRNA regulation, MicF-ompF, intrinsic plasmid, translational fusion

## Abstract

*Yersinia pestis*, which is the causative agent of plague, has acquired exceptional pathogenicity potential during its evolution from *Y. pseudotuberculosis*. Two laterally acquired plasmids, namely, pMT1 and pPCP1, are specific to *Y. pestis* and are critical for pathogenesis and flea transmission. Small regulatory RNAs (sRNAs) commonly function as regulators of gene expression in bacteria. MicF, is a paradigmatic sRNA that acts as a post-transcriptional repressor through imperfect base pairing with the 5′-UTR of its target mRNA, *ompF*, in *Escherichia coli*. The high sequence conservation and minor variation in the RNA duplex of MicF-*ompF* has been reported in *Yersinia*. In this study, we utilized super-folder GFP reporter gene fusion to validate the post-transcriptional MicF-mediated regulation of target mRNA *ompF* in *Y. pestis*. Unexpectedly, upon MicF overexpression, the slightly upregulated expression of OmpF were found in the wild-type strain, which contradicted the previously established model. Interestingly, the translational repression of *ompF* target fusions was restored in the intrinsic plasmids-cured *Y. pestis* strain, suggesting intrinsic plasmids influence the MicF-mediated translational repression of *ompF* in *Y. pestis*. Further examination showed that plasmid pPCP1 is likely the main contributor to the abolishment of MicF-mediated translational repression of endogenous or plasmid-borne *ompF*. It represents that the possible roles of intrinsic plasmids should be considered upon investigating sRNA-mediated gene regulation, at least in *Y. pestis*, even if the exact mechanism is not fully understood.

## Introduction

The human pathogenic species of *Yersiniae* include *Yersinia pestis, Y. pseudotuberculosis*, and *Y. enterocolitica* (Perry and Fetherston, 1997). *Y. pestis* evolved from its ancestor *Y. pseudotuberculosis* several thousands of years ago (Achtman et al., 1999). Interestingly, the two species induce remarkably different diseases by distinct transmission routes. *Y. pestis* causes pandemics of bubonic or pneumonic plague, which is a fatal disease to rodents and humans, and is transmitted by flea biting, whereas *Y. pseudotuberculosis* only causes a chronic and relatively mild disease as an enteric pathogen (Zhou et al., 2006). Although different plasmid combinations exist among *Y. pestis* strains, typical strains of *Y. pestis* harbor three virulence plasmids (pCD1, pMT1, and pPCP1), which encode various virulence determinants. The plasmid pCD1 is commonly shared in all three pathogenic species of *Yersiniae*. The other two plasmids (pPCP1 and pMT1) are laterally acquired by *Y. pestis* (Hu et al., 1998). These three plasmids in *Y. pestis* possess different replication systems. The replicon of plasmid pCD1 belongs to IncFIIA replication system (Perry et al., 1998). Plasmid pPCP1 has a ColE1-like replicon (Hu et al., 1998). The replication region of the pMT1 consists of a structural gene (*repA*) and accessorial elements of replication (Lindler et al., 1998). Although the plasmid-encoded virulence determinants are well documented, the way by which the laterally acquired plasmids have complicated the regulatory networks of *Y. pestis* during its evolution remains ambiguous.

Small RNAs (sRNAs) are involved in regulatory networks as regulators of gene expression to facilitate the quick adjustment of bacterial cells to environmental stresses (Waters and Storz, 2009). *Trans*-encoded sRNAs, which represent a major class of sRNAs in bacteria generally activate or repress mRNA translation by limited base-pairing with mRNA (Desnoyers et al., 2013). The RNA-binding protein Hfq is usually required to help the sRNA-mRNA interaction and RNA stability (Vogel and Luisi, 2011). Hfq has been implicated in stress adaptation and virulence in many bacterial pathogens such as *Y. pestis* (Sittka et al., 2007; Kulesus et al., 2008; Geng et al., 2009; Chiang et al., 2011). MicF is defined as a canonical *trans*-encoded sRNA that regulates outer membrane protein F (OmpF) synthesis in *Escherichia coli* and other related bacteria (Andersen et al., 1989). Approximately 20 nt of MicF forms a perfect RNA duplex by directly pairing with the Shine-Dalgarno region of the *ompF* mRNA. This process occludes the initiation of the 30S ribosome; thus translation is inhibited and the cleavage of mRNA is possibly induced (Andersen et al., 1989). MicF is a highly conserved sRNA in closely related *Enterobacteriaceae* genomes (Delihas, 1997). A strong phylogenetic relationship is also found in MicF/*ompF* interacting sites and RNA duplex in *Yersiniae* (Delihas, 2003). Thus, the regulatory outcome is expected to fit the generalized model, in which MicF forms a duplex with the 5′ UTR of the *ompF* mRNA thereby inhibiting the translation and promoting degradation of the *ompF* transcript. In this study we utilized super-folder GFP reporter gene fusion to validate the MicF-mediated regulation of the *ompF* 5′ UTR in *Y. pestis*. Unexpectedly, the OmpF translation was slightly induced by overexpressed MicF instead of being inhibited in *Y. pestis* Microtus strain 201. This phenomenon is paradoxical to the previous prediction. Interestingly, the translational repression was restored in the intrinsic plasmids-cured strain. Further investigations were conducted to determine which plasmid(s) are responsible for the abrogation of MicF-mediated *ompF* regulation.

## Materials and methods

### Bacterial strains and growth

The bacterial strains in this study are listed in Table 1. *Y. pestis* strain 201 (F1^+^, VW^+^, Pst^+^, and Pgm^+^) isolated from *Microtus brandti* in Inner Mongolia, China, belongs to biovar *Microtus*. This strain is avirulent to humans but highly lethal to mice (Zhou et al., 2004). Strain 201 has gene content that is almost identical to that of *Y. pestis* strain 91001, which possesses four plasmids, namely, pCD1, pMT1, pPCP1, and pCRY1 (Song et al., 2004). All the combinations of plasmid(s)-cured strains derived from strain 201 were constructed by Bin et al. based on the plasmid incompatibility in our laboratory (Ni et al., 2008). *E. coli* and *Y. pestis* were grown to exponential phase on LB (Luria–Bertani) agar with 0.1% arabinose at 37°C or 26°C. Approximate concentrations of antibiotics (100 μg/mL ampicillin and 34 μg/mL chloramphenicol) were added to LB agar throughout this study.

**Table 1:** **Bacterial strains, plasmids, and oligonucleiotides used in this study**.

**Name**	**Characteristics**	**Sources**
**STRAINS**		
MG1655	*E. coli* serotype K12, strain MG1655	Wang's Lab
201	*Y. pestis* wild-type strain 201 (pCD1^+^, pMT1^+^, pPCP1^+^, and pPCRY1^+^)	Song et al., 2004
201-null	*Y. pestis* strain 201 derivative (pCD1^−^, pMT1^−^, pPCP1^−^, and pPCRY1^−^)	Ni et al., 2008
201-pCD1^+^	*Y. pestis* strain 201 derivative (pCD1^+^, pMT1^−^, pPCP1^−^, and pPCRY1^−^)	Ni et al., 2008
201-pMT1^+^	*Y. pestis* strain 201 derivative (pCD1^−^, pMT1^+^, pPCP1^−^, and pPCRY1^−^)	Ni et al., 2008
201-pPCP1^+^	*Y. pestis* strain 201 derivative (pCD1^−^, pMT1^−^, pPCP1^+^, and pPCRY1^−^)	Ni et al., 2008
201-pCD1^+^pMT^+^	*Y. pestis* strain 201 derivative (pCD1^+^, pMT1^+^, pPCP1^−^, and pPCRY1^−^)	Ni et al., 2008
201-pCD1^+^pPCP1^+^	*Y. pestis* strain 201 derivative (pCD1^+^, pMT1^−^, pPCP1^+^, and pPCRY1^−^)	Ni et al., 2008
201-pMT^+^pPCP1^+^	*Y. pestis* strain 201 derivative (pCD1^−^, pMT1^+^, pPCP1^+^, and pPCRY1^−^)	Ni et al., 2008
**PLASMIDS**		
pXG10-SF	A low-copy translational fusion vector with pSC101 origin	Corcoran et al., 2012
pXG-1	Modified pXG10-SF in which sfGFP expression is P_LtetO_-controlled	This study
pXG-OmpF::gfp	OmpF::GFP fusion plasmid by inserting a DNA fragment amplified by primer ompF/R into pXG10-SF	This study
pBAD/HisA	A high-copy expression vector	Invitrogen
pBAD-TF	An inducible transcriptional fusion vector modified from pBAD/HisA	This study
pBAD-MicF	MicF expressing plasmid by inserting a DNA fragment amplified by primer micF/R into pBAD-TF	This study
**Name**	**Sequence (5**′**−3**′**)**	
**OLIGONUCLEIOTIDES**		
ompF-F	TGGATGCATACACAGACGACACCAAACTC	
ompF-R	CTTGCTAGCGGCTAACAGAGCTGGGATTAC	
pBAD-F	TCTGCAGAGCTCGGTACCAAGCTTGCCTGGCGGCAGTAGCGCGGTGGTCCCAC	
pBAD-R	TTGGTACCGAGCTCTGCAGAATTCTATGGAGAAACAGTAGAGAGTTGCGATAAAAAGCG	
pXG-1-F	GAGGGGAAATCTGATGGCTAGCGGATCCGCTGGCTCCGCTGCTGG	
pXG-1-R	CATCAGATTTCCCCTCATGCATGTGCTCAGTATCTCTATCACTGATAG	
micF-F	GTGGAATTCGCTATCATCATTATTTTCCTATCATTGTGG	
micF-R	CATGGTACCTATTCAACTTGAAGTATGACGGGTATAAC	

### Construction of *gfp* reporter fusions plasmid

Plasmid pXG10-SF, which is an improved gfp-based translational fusion vector with the constitutive PLtetO promoter (Corcoran et al., 2012), was used to construct the GFP reporter fusion to *ompF*. An amplicon containing the 5′ UTR of the AUG start codon (92 nt) and 16 amino acids of OmpF was obtained by using the primer pair ompF-F/R. The resulting fragment was fused to sfGFP by inserting it into the *Nsi*I/*Nhe*I-digested pXG10-SF, which yielded the plasmid pXG-*ompF*::*gfp*.

### Construction of sRNA overexpression plasmid and pXG-1

QuikChange® Lightning Site-Directed Mutagenesis Kit (Stratagene) was used to construct an inducible transcriptional fusion vector by modifying an expression vector, pBAD/HisA. According to the manufacturer's instructions, the pBAD/HisA plasmid was amplified with pBAD-F/R primer pair by PCR followed by *Dpn*I treatment. The resulting plasmid pBAD-TF removed the 327-nt fragment containing several elements (RBS, AUG, and polyclonal sites) and introduced *EcoR*I and *Hind*III restriction sites upstream of the *rrnB* terminator sequence. The modified vector pBAD-TF was used as pBAD control. To construct the MicF overexpression vector, a DNA fragment spanning full-length (85 nt) and 58 nt downstream of MicF was ligated to modified pBAD vector and pBAD-MicF was obtained.

The similar protocol was also used to construct a control plasmid designated as pXG-1 with sfGFP expression controlled by PtetO by modifying pXG10-SF. The pXG10-SF plasmid was amplified with primer pair pXG-1-F/R. The resulting plasmid removed the 725-nt *lacZ*-containing fragment between *Aat*II and *Bhe*I and introduced RBS and AUG sequence (GAGGGGAAAUCUGAUG) upstream of *lacZ* from the transcriptional fusion vector pRW50 into the same site.

### Imaging and quantitative measurements of GFP fluorescence

Images of bacteria expressing plasmid-borne *gfp* fusions and grown overnight on LB plates were taken using a CCD camera in Gel Doc XR^+^ image analyzer (Bio-Rad) under the SYBR Green mode. The aliquots of each strain were scraped from the plates and resuspended into PBS buffer. Cell densities were adjusted to OD_620nm_ ≈ 1.5 and 200 μL of bacterial suspensions were placed into 96-well microtiter plates. Two independent cultures as biological replicates and three aliquots as technical replicates were used throughout the study for each strain. Green fluorescence images were captured by SpectraMax M2 MicroplateReaders (Molecular Devices) with an excitation/emission wavelength of 485/525 nm. Fold changes in the MicF-mediated OmpF expression were calculated by dividing the specific fluorescence of strains with MicF overexpression by that of strains with negative control plasmid. The data were analyzed using One-Way analysis of variance (ANOVA) and Sidak's multiple comparisons test, where *P*-values of < 0.01 were considered significant.

### OmpF detection by using western blot

*Y. pestis* were grown in LB agar plate at 26°C for 36 h. Equal amounts of bacterial aliquots were collected and lysed by ultrasonication. Total cellular proteins were separated on SDS-PAGE and immunoblotted with anti-OmpF multiclonal antibody and DyLight 680–labeled goat anti-rabbits antibody followed by detection using the Odyssey Infrared Imaging System. The abundance values were calculated as the expression level of the derivatives divided by that of strain 201 using the Quantity One software. The GroEL protein was detected in parallel as control.

### RNA detection by using northern blot

Total RNA was then extracted from various bacterial strains grown in LB agar plate in the presence of 0.1% arabinose at 26°C for 36 h using the TRIzol Reagent (Invitrogen). Total RNA samples (1 μg) were denatured at 70°C for 5 min, separated on 6% polyacrylamide 7 M urea gel, and transferred onto Hybond N+ membranes (GE) via electroblot. The membranes were UV-crosslinked and pre-hybridized for 1 h. Northern hybridization was performed by adding the DIG-labeled MicF-specific RNA probe synthesized by *in vitro* transcription using T7 RNA polymerase. The RNAs were immunologically detected according to the instructions on the DIG Northern Starter Kit (Roche). Band intensities on the Northern blots were quantified by Quantity One software. The 5s rRNA species was monitored in parallel as control.

## Results and discussion

In this study, the MicF-mediated *ompF* regulation was validated using a two-compatible-plasmid reporter system established by (Corcoran et al., 2012). The sRNA plasmid is a high-copy vector (pBAD), which ensures the high level of expressed MicF and minimizes the inference of chromosome-encoded MicF. The target plasmid is a low-copy vector (pXG10), where transcription of the *ompF::gfp gene* is driven by the constitutive promoter P_LtetO_, thereby uncouples the translation from *ompF* transcription. In this system MicF is overexpressed and *ompF*-*gfp* fusion represents the OmpF abundance. We found that the intrinsic plasmid(s) have an effect on sRNA-mediated regulation, at least on the MicF-mediated *ompF* translation.

### Intrinsic plasmids accounted for the abolishment of the MicF-mediated translational repression of *ompF* in *Y. pestis*

*Y. pestis*-specific *ompF*-*gfp* fusion and sRNA MicF were cloned into low- and high-copy vectors, respectively. Both plasmids were transformed into *E. coli* and *Y. pestis*. All the plasmids were checked throughout all the tested strains by PCR. The results showed that all the plasmids (pMT1, pCD1, pPCP1, and MicF-expressing plasmid pBAD-MicF) were present as expected (Figure S1). The reporter GFP fluorescence activity was monitored to evaluate the regulatory roles in various bacterial strains.

**Figure 1:**
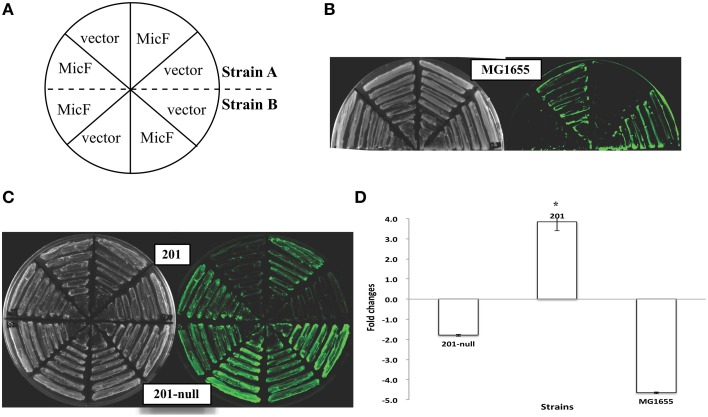
**Expression of the ***ompF::gfp*** fusions upon MicF overexpression in ***Escherichia coli*** and ***Yersinia pestis*** strains**. **(A)** Layout map of bacterial strains carrying the control plasmid without sRNA overexpression (denoted as “vector”) or MicF-overexpression plasmid (denoted as “MicF”). Duplicate images of representative strains are shown in parallel on plates. **(B)** Representative fluorescence images of *E. coli* strain MG1655. The image obtained under visible light mode is shown at the left panel and that of the same plate under the fluorescence mode at the right panel. **(C)** Representative fluorescence images of *Y. pestis* WT strain (201) and its plasmid-cured derivative strain (201-null). **(D)** Quantitative measurements of fluorescence produced by the tested strains. Fold changes are provided as the ratio of fluorescence values detected in MicF-overexpressed bacterial strains divided by those detected in strains carrying pBAD blank vectors. Values presented are means ± standard deviations of two independent experiments. The asterisks indicate statistically significant differences compared to the values detected in strain MG1655.

**Figure 2:**
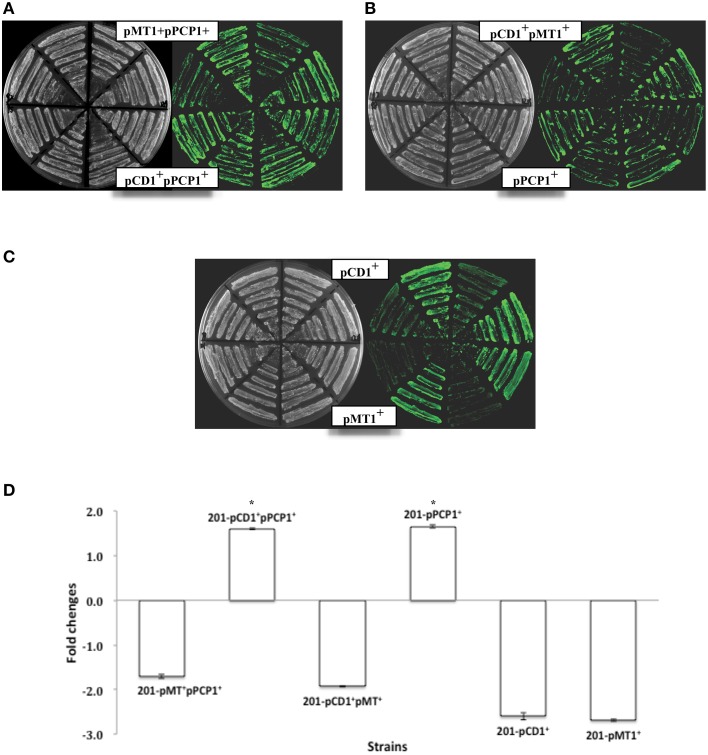
**Expression of the ***ompF::gfp*** fusions upon MicF overexpression in the ***Yersinia pestis*** strains with different plasmid combinations**. Representative fluorescence images of *Y. pestis* derivative strains are shown **(A–C)**. The corresponding quantitative results are also shown **(D)**, in which the asterisks indicate statistically significant differences compared to the values detected in strain MG1655 shown in Figure 1.

The inhibitory translation of *ompF-gfp* fusion upon MicF overexpression (approximately five-fold repression) was observed in *E. coli* strain MG1655. Only the 1.8-fold repression was observed in the *Y. pestis* strain 201 that is cured of all the endogenous plasmids (Figure 1). This finding is consistent with the results previously reported on *E. coli* (Mizuno et al., 1984) and also confirms that MicF-mediated *ompF* repression occurs in the 5′-UTR. Interestingly, the inhibitory effect was not found in the *Y. pestis* WT strain 201. Instead, more than three-fold upregulation of OmpF-GFP expression was observed under the same conditions (Figure 1). The relative quantification of fluorescence value was also measured in *Y. pestis* strains with different combinatorial plasmids grown to exponential phase in LB medium followed by arabinose induction for 1 h. Similar tendency was found in *Y. pestis* strains grown in liquid medium as that found in solid medium (Figures 1, 2 and Figure S2). To exclude the possibility that the effect was caused by the translational reporter system, we construct plasmid pXG-1 which sfGFP expression is constitutively P_LtetO_-controlled. No changes in fluorescence intensity were found between pBAD-MicF and pBAD-vector groups in *Y. pestis* strain 201 and 201-null carrying pXG-1 (Figure S3). The different regulatory consequences in *Y. pestis* strains carrying or cured of plasmids indicated that the virulence-associated plasmids might have been recruited to sRNA-mediated regulatory networks in the chromosome during evolution.

### Plasmid pPCP1 alone abolished the MicF-mediated translational repression of *OmpF*

*Y. pestis* strains carrying different combinations of the three plasmids (pMT1, pPCP1, and pCD1) were used to determine the probable plasmid(s) responsible for the abolished MicF-mediated regulation of *ompF*. Upon MicF overexpression, *ompF* translation remained repressed in the pCD1- or pMT1-containing strains, as observed in the plasmid-null strain. However, the expression level of OmpF-GFP fusion protein was even slightly upregulated about 1.6-fold in the *Y. pestis* strain carrying plasmid pPCP1. Such activation was also observed in the strain carrying both plasmids pPCP1 and pCD1 (Figure 2). Plasmid pPCP1 alone could overwhelm the translational repression in the plasmid-cured strain, thereby suggesting that pPCP1 may mainly contribute to the abolishment of sRNA-mediated regulation. Paradoxically, translational repression was still found in the strain with pPCP1 and pMT1. We speculated that addition of pMT1 might interfere with the effect of pPCP1 on sRNA-mediated regulation because of the potential interactions between these two plasmids.

**Figure 3:**
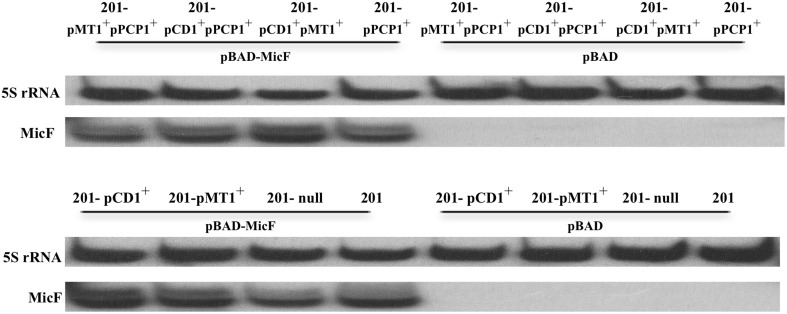
**Detection of MicF expression in various strains of ***Y. pestis*****. MicF expression detected by Northern Blot, in which 5S rRNA images from each tested strain were provided as control.

We also monitored MicF in the different bacterial strains under the same conditions as those shown in Figures 1, 2. No MicF were found expressed in the pBAD control of various *Y. pestis* strains by using Northern Blot, which might be mainly due to huge disparity in copy number of MicF between plasmid pBAD and chromosome and/or low expression levels of endogeneous MicF under our experimental conditions. Additionally, no significant differences in MicF overexpression were found among *Y. pestis* strains (Figure 3).

### Validation of MicF-mediated regulation of endogenous *OmpF* elicited by intrinsic plasmids

**Figure 4:**
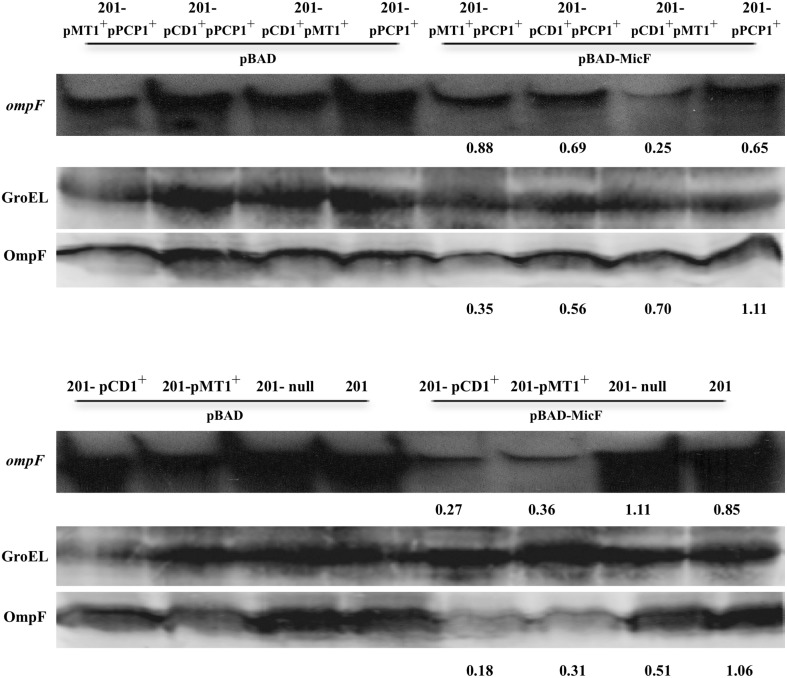
**Abundance detection of endogenous ***ompF*** transcript and OmpF protein in various strains of ***Y. pestis*****. Northern Blot was used to detect the chromosome-encoded *ompF* transcript in various strains of *Y. pestis* grown under the same conditions as those shown in Figure 3. Meanwhile, the anti-OmpF rabbit multiclonal antibody was used in Western Blot to detect the endogenous OmpF protein in the indicated strains, in which GroEL protein images from each strain were provided as control. The numbers indicated below each panel represent the fold changes of mRNA or protein abundance detected in strains carrying pBAD-MicF divided by that of the corresponding strains carrying pBAD control vector.

To test whether the intrinsic plasmids interfere with the MicF-mediated regulation of the chromosome-encoded target gene *ompF*, the abundances of endogenous *ompF* transcript and OmpF protein were validated in various strains of *Y. pestis* by Northern Blot and Western Blot, respectively (Figure 4). In agreement with the results of gene fusion reporter systems, the abundance of *ompF* transcript or OmpF protein were found decreased 2.0–5.0 fold in four pPCP1-cured strains (122-null, 122-pCD1^+^pMT1^+^, 201-pCD1^+^, and 201-pMT1^+^) upon MicF overexpression relative to that in the control strains. Strikingly, the *ompF* transcript was stable, but approximate three-fold decrease was found in the expression level of OmpF in the strain 122-pMT1^+^pPCP1^+^, which is also consistent with the findings presented in Figure 2. However, only the comparable levels of *ompF* and OmpF were found among three groups of pPCP-containing strains (122, 122-pPCP1, and 122-pCD1^+^pPCP1^+^). The downregulation phenomenon is roughly consistent with that of plasmid-borne *gfp* fusion. The discrepancy is likely due to the different sensitivity between translational fusion assay and Western blot. However, no obvious upregulation was found in strain 201. Maybe the actual activation was magnified by translational fusion assay or veiled by detection threshold of Western blot or Northern Blot. Taken together, this observation further confirmed the conclusion that intrinsic plasmids have the potential impacts on abolishment of MicF-mediated *ompF* regulation in *Y. pestis*.

Our study demonstrated that the mobile elements affect sRNA-mediated regulation in *Y. pestis*. Distinct conclusions may be drawn if various strains carrying different plasmids are used to investigate the sRNA-mediated regulation. For example, Hfq reportedly represses the biofilm formation in *Y. pestis* KIM6+ (an avirulent derivative of the fully virulent strain KIM, which was cured of the pCD1 plasmid) grown in BHI medium (Bellows et al., 2012). Opposite effects on biofilm formation were observed in the *hfq* mutant of *Y. pestis* wild-type strain 201 and its derivative strain lacked the plasmid pCD1 (unpublished data). Therefore, the background bacteria to be used as control strain should be carefully selected. The possible roles of plasmids in gene regulation should be considered even if the exact mechanism is not fully understood. Although a relationship exists between the intrinsic plasmids and sRNA-mediated regulation, the presence or absence of any plasmid did not cause the clear-cut effects on MicF-mediated *ompF* regulation. This phenomenon may indicate that mutual interactions exist among intrinsic plasmids in *Y. pestis*, and such interactions further influence sRNA-mediated regulation.

### Conflict of interest statement

The authors declare that the research was conducted in the absence of any commercial or financial relationships that could be construed as a potential conflict of interest.
