# Supplementary figures and images for: Intrinsic plasmids influence MicF-mediated translational repression of ompF in Yersinia pestis
Source: Front Microbiol. 2015 Aug 21;6:862. doi: 10.3389/fmicb.2015.00862 (PMC4543863; doi:10.3389/fmicb.2015.00862)

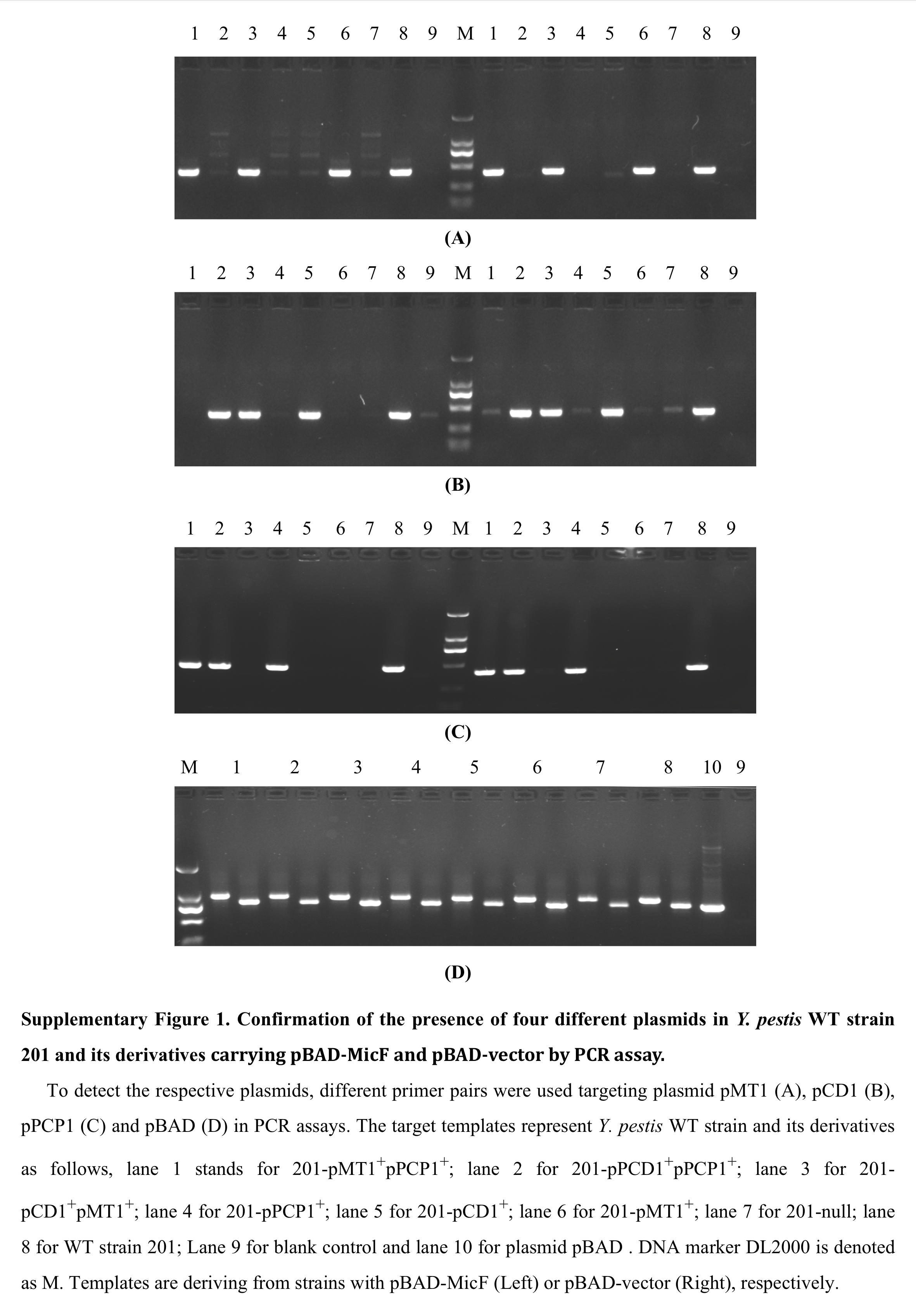

Supplement: Supplementary file 1 [file Image1.TIFF]

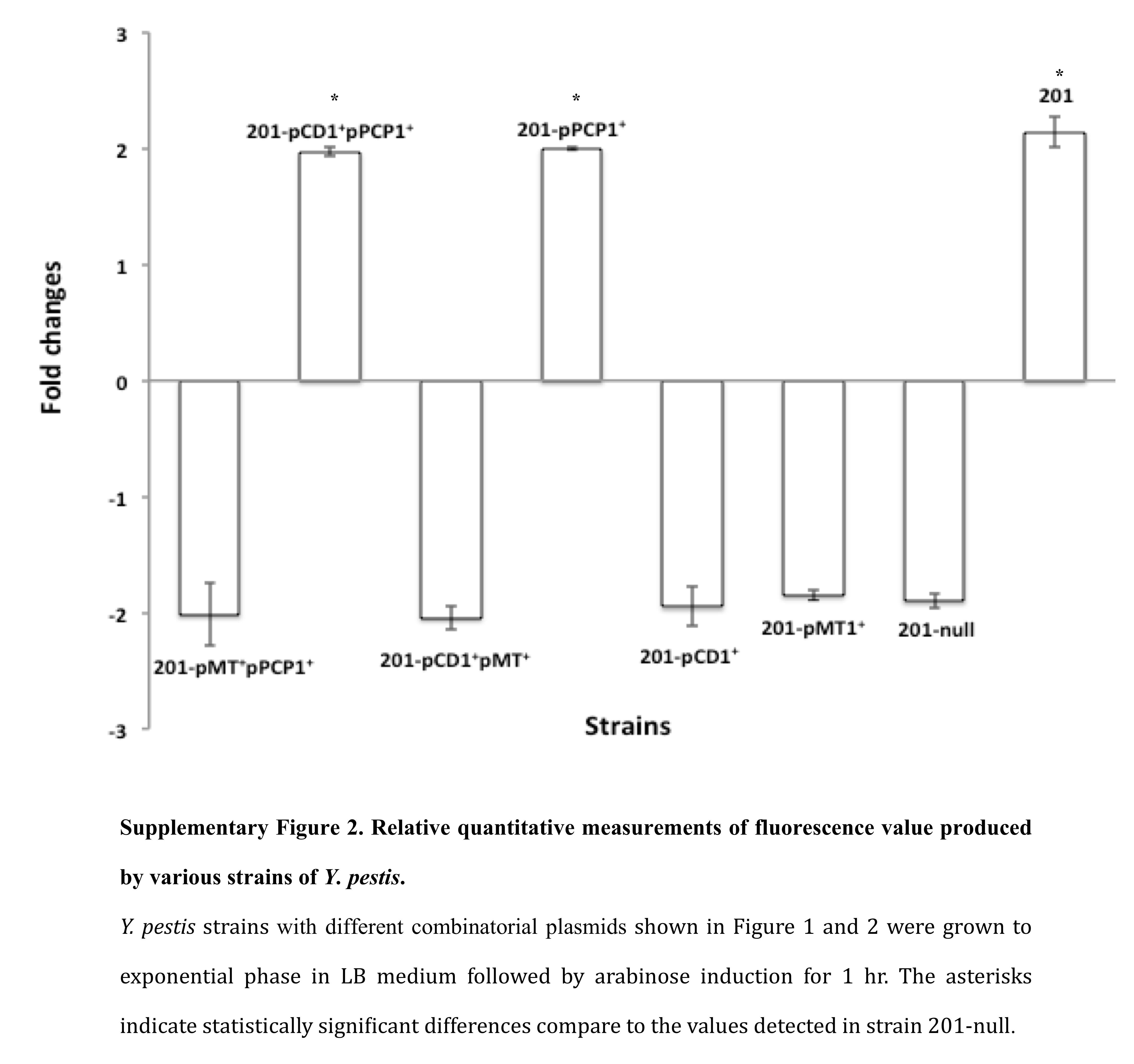

Supplement: Supplementary file 2 [file Image2.TIFF]

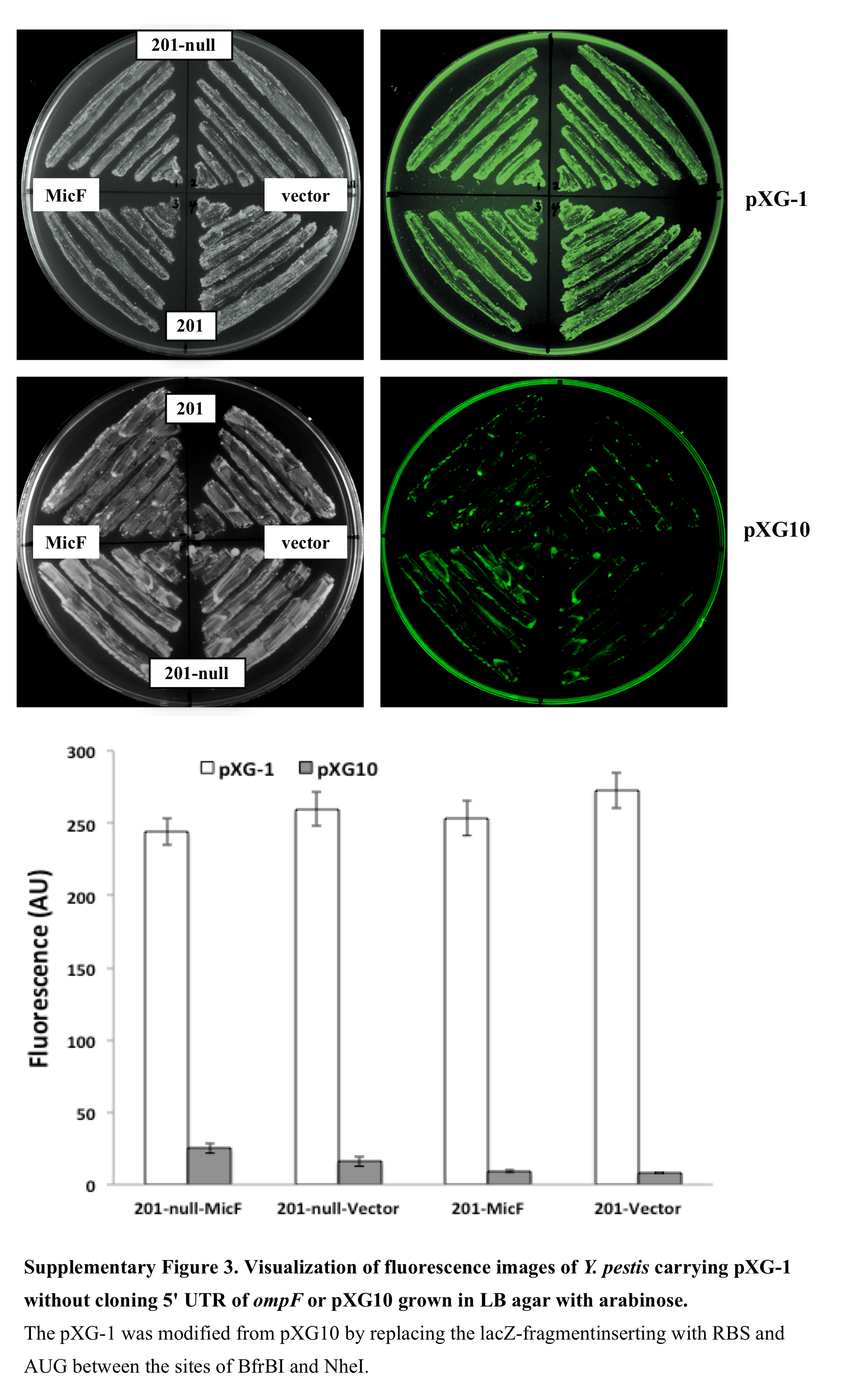

Supplement: Supplementary file 3 [file Image3.TIFF]
